# Epilogue: The Language Opportunities for the 21st Century

**DOI:** 10.1177/0261927X20933997

**Published:** 2020-09

**Authors:** Margaret J. Pitts

**Affiliations:** 1 University of Arizona, Tucson, AZ, USA

**Keywords:** positive psychology, positive social science, positive inquiry, positive communication, language attitudes, intergroup communication, linguistic diversity, COVID-19

## Abstract

This epilogue to the Special Issue on *Language Challenges in the 21st Century* offers commentary on the current state of social scientific inquiry in the field of language and social psychology. Inspired by the seven articles that make up this Special Issue, I became curious about what we would find if we sought language opportunities instead of language challenges in the 21st century. I recommend future scholarship at the intersections of global and linguistic diversity include a positive social science approach in order to consider the full spectrum of challenges and assets. I conclude with a note about the direction of future research related to COVID-19.

To close this Special Issue on Language Challenges in the 21st Century, I point to the guest editors’ promise to rouse curiosity in examining the implications of increasing global diversity and linguistic intersections for human relations.^1^ I also rise to meet their entreaty to provide an additional perspective to the present set of *challenges* by insisting that we also consider the equally present *potentialities*. Consistent with the values and aims of the *Journal of Language and Social Psychology*, the articles in this Special Issue report on (and from) multilingual and multicultural contexts. The multimethodological and interdisciplinary perspectives represented in this issue offer considerable insight into the challenges surrounding language in the 21st century from many different angles. The Special Issue meets the challenge of rousing intellectual and moral curiosity and makes a valuable contribution to contemporary and future scholarship. Yet, centering the point of inquiry on language *challenges* limits the potential for exploration. What about language opportunities?

Positive psychology, the “psychology of positive human functioning,” and similar movements in related fields, such as positive communication (Pitts & Socha, 2013; Socha & Pitts, 2012), emerged in response to the predominant focus in the social and behavioral sciences on pathology and problems (Seligman & Csikszentmihalyi, 2000). Positive social science is the science of human flourishing and the promotion of well-being—“what is going right.” Yet, the focus on “what is going wrong” remains pervasive in social science and structures the very way we approach scientific inquiry and the questions we ask. Turning to this Special Issue, what happens when we apply a positive social science lens and ask not what are the language challenges of the 21st century, but rather what are the opportunities?

My goal here is to introduce additional avenues for examining language in the 21st century from a positive social science perspective that stem from articles in this Special Issue. In their prologue, Birney, Roessel, Hansen, and Rakić (2020) group the seven articles into three overarching themes highlighting the core relationships among the articles as they address language challenges. I adopt the same structure here with the goal of shining light on the positive potential within each area in hopes that it also inspires curiosity to explore new areas of language research.

## The Power of Language to Form and Perpetuate Stereotypes

The first two articles in the Special Issue demonstrate the power of negative and comparative framing. Burgers and Beukeboom (2020) show the damaging impact of using generic labels and of using negation in affirmative behavioral descriptions about an unknown individual’s competence (“not stupid” vs. “smart”). The perceived competency of an individual labeled “not stupid” was diminished compared to when the individual received positive affirmation. The mere presence of negation even when used as a “positive” casts a shadow over the interpretation. There is a difference between affirming, or actively pursuing positive communication, and “not disaffirming.” A positive social science lens might set the focus on the power of specific labels and of affirmations in message framing.

Bruckmüller and Braun (2020) point to a similarly distressing language habit involving metaphor and the tendency to focus on women’s underrepresentation rather than men’s overrepresentation in leadership. Their discussion inspired me to think further about ways in which novel metaphors can break down barriers by shifting our attention to the implicit bias present in metaphor. Messages that are novel or uniquely formulated may trigger mindful (rather than mindless) attention, thereby increasing individuals’ capacity to attend to unique information or implied relationships (Langer et al., 1978). Instead of invoking glass ceiling metaphors (for women), why not use cloud ceiling metaphors (for men)? The authors conclude that novel metaphors may bring attention to language norms that perpetuate gender inequality allowing individuals to first recognize the inequality perpetuated in language and then to act to change it. Exploring this phenomenon from a mindful communication perspective could be fruitful.

With regard to these two articles, one avenue for future exploration could be found in the power of micro-affirmations in daily discourse. In a time where places of work, education, and leisure are concerned with “micro-aggressions” (Sue, 2010) or “micro-inequalities,” the concept of “micro-affirmations” has emerged to bring equal attention to the myriad of ways we use language subtly to affirm each other. Micro-inequalities are described by Rowe (2008) as small linguistic and behavioral events that are difficult to prove, often covert, and which point toward difference in ways that continuously diminish the individual. Alternatively, micro-affirmations refer to equally small linguistic and behavioral acts that are difficult to prove, often covert, and yet serve to bolster individuals, occurring “wherever people wish to help others to succeed” (Rowe, 2008, p. 46). One potential to overcome systemic inequalities perpetuated through language is to work purposively to create a micro-affirmational culture (Molina et al., 2019)—an intentional practice.

## Language as Integral to Intersectionality and Combined Identities

The next two articles in the Special Issue contribute to our knowledge about the intersectionality of language and social identities highlighting the complex relationships between accent, nationality, language use, stereotypes, and language attitudes. Indeed, they direct our attention to the difficulties and negative outcomes resulting from our lack of intersectional awareness. Birney, Rabinovich, and Morton (2020) argue language *itself* is intersectional, revealing, concealing, and suggesting multiple personal and social identities at once including age, gender, regional identity, social status, and many others. And yet, Rakić et al. (2020) present findings that suggest that despite increased identity intersectionality, individuals, at a very basic level, may not have the tools or the motivation to recognize and value the distinctiveness of others. At the same time, the two articles show that individual perceptions *are* influenced by the unique intersections of language and social identity. Specifically, Birney, Rabinovich, and Morton (2020) found that in national minority group contexts, it was the combination of national identity and nonnative speaker accent, more so than accent or national identity alone, that had important implications for interpersonal and intergroup perceptions.

The focus on the challenges related to intersectionality raised my curiosity to ask, what can a positive lens put into focus with regard to linguistic diversity? One possibility is to promote mindfulness practices to increase appreciation for and awareness of diversity. In my own work, I have suggested that if we demonstrate appreciation for the sounds and variations of language—to savor aesthetic forms of communication (Pitts, 2019)—we can extend that appreciation to linguistically diverse individuals and communities. MacIntyre’s (2016) work in the positive psychology of second language acquisition also comes to mind wherein second language teachers create opportunities for learners to flourish by capitalizing on positive emotions, flow, and character strengths—instilling appreciation for language learning and application. The positive social science perspective puts into focus additional questions: What are the intersectional identities that contribute to flourishing? How do individuals manage identity needs and identity gaps to cultivate one’s higher self? And, from the point of view of the scholarship, to what extent does intersectionality necessitate attending to, and spotlighting, distinctive components and not the whole? Whose values (or questions) are served when we do?

## The Role of Language in Shaping Interpersonal and Intergroup Relations

The final set of articles broadly asks, what threats, but also what protections does one’s language, and access to multiple languages, provide. These articles inspired me to think about the right to pursue a meaningful life in the language of one’s heart and home. Although The Universal Declaration of Human Rights, established by the United Nations General Assembly in 1948 (United Nations, n.d.), falls short of establishing the right to speak in one’s home language, the Declaration does lay the foundation for such an endeavor. Specifically, Article 2 recognizes that “everyone is entitled to all the rights and freedoms set forth in this Declaration, without distinction of any kind, such as race, colour, sex, *language*, religion, political or other opinion, national or social origin, property, birth or other status.” Article 19 establishes the right to freedom of expression. Article 22 establishes the right to social security and to the cultivation or realization of “social and cultural rights indispensable for [their] dignity and the free development of [their] personality,” which I read to include the right to pursue a meaningful life in one’s language of ancestry. Almost 60 years later, the UN General Assembly adopted a resolution on multilingualism which recognizes “multilingualism as a means of promoting, protecting and preserving diversity of languages and cultures globally [and] also that genuine multilingualism promotes unity in diversity and international understanding” (United Nations, 2007, p. 1). The final three articles put into focus the benefits of multilingualism and of having access to a heritage language including individual and community resilience.

In their article, Klar et al. (2020) identify the positive potential for code-mixing (e.g., signaling bilingual proficiency, modernity, and social mobility) and yet find and focus their attention on the negative evaluations of code-mixing among Arab-Palestinians in Israel. Still, code-mixing persists and even flourishes in some communities with numerous benefits. A positive social scientific perspective could put into focus the relationships between sociocultural resilience, flourishing, and code-mixing. What would happen if influential community members in governmental, education, religious, and media sectors promoted and authenticated code-mixing?

Jelić et al. (2020) investigate implications of practicing the right to pursue education in one’s native tongue in Croatia. The authors take a close examination of the factors that contribute to perceptions of the minority language use as a threat and the resulting prejudicial and discriminatory acts. Their findings point to ethnonationalism, and not ethnic identity, as detrimental to intergroup relations within this context. The authors also recognize that due to differing group histories, engaging in the acts of everyday living (i.e., pursuing education in this case) in one’s home language might *not* result in intergroup tension. Just as “not” being sick is not the same as “being healthy,” “not” resulting in intergroup tension is not the same as resulting in community benefits. Traditional intergroup framing implicitly directs our attention toward conflict, competition, and disharmony, but there must be room for a positive social scientific approach to intergroup relationships. This article inspired me to ask whether there was something more than just “not” resulting in intergroup tension—intergroup harmony or flourishing, for example. How is the pursuit of education in one’s home language for these minority groups a boon to community and self?

Finally, I come to Skrodzka et al.’s (2020) contribution which points at the potential for language to serve as a protective factor in the context of deep historical trauma. Similar to Ladegaard’s (2018) body of work on the potential use of language as a shield against painful disclosures of abuse among migrant domestic laborers, this work compelled me to think about how individuals use language, at times, to emphasize and soak-in an experience (even a negative one) and at other times use language as a shield to distance oneself from an experience. Thus, in addition to the unique challenges it brings, having access to multiple ways of experiencing and expressing our world through language can also be an asset in the face of trauma. Does multilingualism, and do the communities that support multilingualism, engender a unique type of human resilience and empathy?

## Final Thoughts

The articles in this Special Issue inspired me to think beyond the box of social scientific inquiry. As social scientists, are we hindering our pursuit of knowledge by holding on to shibboleths about established instruments and measures that often assess only the limitations of human experiences and not its full potential (e.g., measuring intergroup tension, but not harmony)? What roads open before us when we “flip” the question and critically ask, “What are the language opportunities of the 21st century?” How can we harness the potential of global intersectionality for the betterment of groups and communities?

The contributions to this Special Issue give insight into the complexities of global intersections and the complications that lie within. The editors of the Special Issue invite us to meet these challenges with curiosity, an open mind, and an open heart. I hope to have further sparked curiosity by posing some questions of my own. I believe the field of language and social psychology, with its particular interest in interpersonal and intergroup relations, has much to gain by adopting a positive social and behavioral science approach.

## Coda

As we consider the most pressing language and social psychology questions of the 21st century, we must acknowledge the challenges and opportunities that face us amid the Covid-19, Coronavirus pandemic. The Coronavirus has halted and altered our social interactions and physical contact. It has made salient some of our more subtle intergroup differences with respect to nationality, age, health, ability, social class, profession, and access to technology, transportation, and other resources. It has made evident the ways in which we are vulnerable and resilient. It has affected the lives and livelihood of all humanity, as well as our ecosystem, in unprecedented and unexpected ways. Thus, in our quest to understand the linguistic, intergroup, interpersonal, and health impacts of COVID-19, I urge my fellow social scientists to pause and consider ways to study the full breadth of the impact of COVID-19, including scholarship dedicated to the exploration of what is going right amid so much of what is going wrong. We must ensure that our questions and tools do not lead us into a boundless chasm of despair, measuring only depression, anxiety, uncertainty, hostility, and mistrust, but also boundlessly measure hope, positivity, a sense of purpose or meaningfulness in one’s life.

A positive social science approach could focus on demonstrations of human strength, virtue, and resilience; the acknowledgement and recognition of others we have seen in community responses to health care workers and grocery clerks; expressions of grace and generosity with self and others as we fumble through learning how to live virtually; awakenings of gratitude for things small and big; and authenticity in social interactions because we are too cognitively overloaded or too confined in one small physical space to manage our front- and back-stage identities. A positive science approach recognizes that this shared global experience creates space for empathy and compassion. It provides opportunity to examine positive intergroup dynamics such as harmony, collaboration, and integration rather than just focus on intergroup conflict or competition.

Language and social psychology scholars can lead the scientific exploration of the protective forces of language in times of uncertainty and turbulence. We can lead the exploration of resilience, resourcefulness, and creativity that draws humans together in novel ways despite orders to stay at home. We can lead the exploration of individual and community assets that contribute to flourishing, thriving, and persistence in the face of grief, loss, and disruption. It is time to look for what is going right. It is my hope that this call to action with regard to COVID-19 research resonates with many readers of the *Journal of Language and Social Psychology.* Please keep an eye out for, and consider submitting work to, the upcoming Special Issue, “Stay Safe and Stay at Home! Research on Language and Communication Related to Corona” guest edited by Regina Jucks (*jucks@uni-muenster.de*) and Friederike Hendriks (*f.hendriks@unimuenster.de*).

## Notes

1. Much of the writing of this epilogue took place prior to the global COVID-19 pandemic, but readers will find in the coda to this epilogue some thoughts about extending a positive social scientific lens to future research related to COVID-19. Now is the time to look for and raise up what is going right.

## References

[bibr1-0261927X20933997] Birney M. B. Rabinovich A. Morton T. A. (2020) Where are you from? An investigation into the intersectionality of accent strength and nationality status on perceptions of non-native speakers in Britain. Journal of Language and Social Psychology, 39(4), 495�515. 10.1177/0261927X20932628

[bibr2-0261927X20933997] Birney M. B. Roessel J. Hansen K. Rakić T. (2020). Prologue: Language challenges in the 21st century. Journal of Language and Social Psychology, 39(4), 428�437. 10.1177/0261927X20933315

[bibr3-0261927X20933997] Bruckmüller S. Braun M. (2020). One group’s advantage or another group’s disadvantage? How comparative framing shapes explanations of, and reactions to, workplace gender inequality. Journal of Language and Social Psychology, 39(4), 457�475. 10.1177/0261927X20932631

[bibr4-0261927X20933997] Burgers C. Beukeboom C. J. (2020). How language contributes to stereotype formation: Combined effects of label types and negation use in behavior descriptions. Journal of Language and Social Psychology, 39(4), 438�456. 10.1177/0261927X20933320

[bibr5-0261927X20933997] Jelić M. Uzelac E. Biruški D. C. (2020) Intergroup threat as a mediator of ethnic identification and intergroup orientations. Journal of Language and Social Psychology, 39(4), 534�550. 10.1177/0261927X20932632

[bibr6-0261927X20933997] Klar Y. Mar’i A. A. Halabi S. Bashir A. Bashir B. (2020). Reactions of Arab-Palestinians in Israel towards an in-group member mixing Hebrew or English with Arabic. Journal of Language and Social Psychology, 39(4), 516�533. 10.1177/0261927X20933657

[bibr7-0261927X20933997] Ladegaard H. J. (2018). Codeswitching and emotional alignment: Talking about abuse in domestic migrant-worker returnee narratives. Language in Society, 47(5), 693-714. 10.1017/S0047404518000933

[bibr8-0261927X20933997] Langer E. J. Blank A. Chanowitz B. (1978). The mindlessness of ostensibly thoughtful action: The role of “placebic” information in interpersonal interaction. Journal of Personality and Social Psychology, 36(6), 635-642. 10.1037//0022-3514.36.6.635;10.1037/0022-3514.36.6.635

[bibr9-0261927X20933997] MacIntyre P. D. (2016). So far so good: An overview of positive psychology and its contributions to SLA. In Gabryś-Barker D. Gałajda D. (Eds.), Positive psychology perspectives on foreign language learning and teaching (pp. 3-20). Springer. 10.1007/978-3-319-32954-3_1

[bibr10-0261927X20933997] Molina R. L. Ricciotti H. Chie L. Luckett R. Wylie B. J. Woolcock E. Scott J. (2019). Creating a culture of micro-affirmations to overcome gender-based micro-inequities in academic medicine (Commentary). American Journal of Medicine, 132(7), 785-787. 10.1016/j.amjmed.2019.01.028 30716299

[bibr11-0261927X20933997] Pitts M. J. (2019). The language and social psychology of savoring: Advancing the communication savoring model. Journal of Language and Social Psychology, 38(2), 237-259. 10.1177/0261927X18821404

[bibr12-0261927X20933997] Pitts M. J. Socha T. (Eds.). (2013). Positive communication in health and wellness. Peter Lang.

[bibr13-0261927X20933997] Rakić T. Steffens M. C. Sazegar A. (2020). Do people remember what is prototypical? The role of accent-religion intersectionality for individual and category memory. Journal of Language and Social Psychology, 39(4), 476�494. 10.1177/0261927X20933330

[bibr14-0261927X20933997] Rowe M. (2008). Micro-affirmations & micro-inequities. Journal of the International Ombudsman Association, 1, 45-48.

[bibr15-0261927X20933997] Seligman M. E. P. Csikszentmihalyi M. (2000). Positive psychology: An introduction. American Psychologist, 55(1), 5-14. 10.1037//0003-066X.55.1.5 11392865

[bibr16-0261927X20933997] Skrodzka M. Hansen K. Olka J. Bilewicz M (2020). The twofold role of a minority language in historical trauma: The case of Lemko minority in Poland. Journal of Language and Social Psychology, 39(4), 551�566. 10.1177/0261927X20932629

[bibr17-0261927X20933997] Socha T. J. Pitts M. J. (Eds.). (2012). The positive side of interpersonal communication. Peter Lang.

[bibr18-0261927X20933997] Sue D. W. (2010). Microaggressions in everyday life: Race, gender, and sexual orientation. Wiley. 10.1007/s10597-018-0245-9

[bibr19-0261927X20933997] United Nations. (2007). Resolution adopted by the General Assembly. 61/266. Multilingualism. http://www.imo.org/en/OurWork/Conferences/Documents/A.RES.61.266.pdf

[bibr20-0261927X20933997] United Nations. (n.d.). Universal declaration of human rights. https://www.un.org/en/universal-declaration-human-rights/

